# Optimizing non-pharmaceutical intervention strategies against COVID-19 using artificial intelligence

**DOI:** 10.3389/fpubh.2023.1073581

**Published:** 2023-02-13

**Authors:** Vito Janko, Nina Reščič, Aljoša Vodopija, David Susič, Carlo De Masi, Tea Tušar, Anton Gradišek, Sophie Vandepitte, Delphine De Smedt, Jana Javornik, Matjaž Gams, Mitja Luštrek

**Affiliations:** ^1^Department of Intelligent Systems, Jožef Stefan Institute, Ljubljana, Slovenia; ^2^Jožef Stefan International Postgraduate School, Ljubljana, Slovenia; ^3^Department of Public Health and Primary Care, Ghent University, Ghent, Belgium; ^4^Leeds University Business School, University of Leeds, Leeds, United Kingdom

**Keywords:** COVID-19, multi-objective optimization, epidemiological modeling, machine learning, intervention plans

## Abstract

One key task in the early fight against the COVID-19 pandemic was to plan non-pharmaceutical interventions to reduce the spread of the infection while limiting the burden on the society and economy. With more data on the pandemic being generated, it became possible to model both the infection trends and intervention costs, transforming the creation of an intervention plan into a computational optimization problem. This paper proposes a framework developed to help policy-makers plan the best combination of non-pharmaceutical interventions and to change them over time. We developed a hybrid machine-learning epidemiological model to forecast the infection trends, aggregated the socio-economic costs from the literature and expert knowledge, and used a multi-objective optimization algorithm to find and evaluate various intervention plans. The framework is modular and easily adjustable to a real-world situation, it is trained and tested on data collected from almost all countries in the world, and its proposed intervention plans generally outperform those used in real life in terms of both the number of infections and intervention costs.

## 1. Introduction

The first line of defense against the spread of the SARS-CoV-2 virus was the introduction of Non-Pharmaceutical Interventions (NPIs) by national governments. With the virus being aerosol-borne, some of the key measures included the use of face masks and restrictions on gatherings, which have often resulted in partial or full lockdowns. While effective at reducing the number of infections (1, 2), restrictive NPIs also presented immense Socio-Economic Costs (SECs) to the population (3). Policy-makers were faced with an almost impossible task of carefully balancing NPI costs against the predicted NPI benefits, largely without having appropriate tools and data for evidence-based decisions.

To add complexity to the problem, in a typical intervention plan adopted by policy-makers, a combination of NPIs would be used, each of them taking place for different periods of time. These plans were usually prepared by expert panels who had the challenge of selecting intervention plans without assurance that they would really flatten the infection curve enough to be lifted within the expected period (4, 5).

While many models for the prediction of daily infections and the impact of NPIs on the spread of the pandemic have been proposed (1, 2), little work has been done regarding the prescription of intervention plans—especially taking into account the NPI costs and how to best combine NPIs. Yousefpour et al. (6), for example, proposed a framework based on SEIRD models and multi-objective optimization to prescribe NPIs. However, the optimization did not operate on real-life NPIs, and as such, this approach cannot be directly used by policy-makers. Chen et al. (7) created a linear programming tool to explore the trade-off between the expected mortality rate of COVID-19 and return to normal activities, while Yaesoubi et al. (8) developed a decision tool to determine when to trigger, continue, or stop physical distancing intervention in order to minimize both the deaths from COVID-19 and intervention duration. Both studies combined the objectives into a single function and the final result was a single intervention plan. Such approaches require a strong predefined preference on how to balance the objectives, which is often difficult to define in practice. In addition, none of the three approaches was extensively tested on various epidemiological scenarios. For this reason, their generalization to real-world situations is not known.

A more structured attempt to research the possibility of using artificial intelligence (AI) to automatically prescribe intervention plans was made by the $500K Pandemic Response Challenge (9), organized by XPRIZE and sponsored by Cognizant. The participants were tasked with finding good trade-offs between the costs of NPIs and their benefits—and assemble three-month intervention plans for each territory (all countries and some sub-country regions). An approach proposed by the sponsor [Miikkulainen et al. (10)] involved the use of evolutionary algorithms to evolve neural networks that prescribe intervention plans. This approach was intended to point the way for the competitors, who would go on to develop better-performing approaches. The competition ended with two “Grand Prize Winners.” One of them (11) combined two prescriptors: the first selected the most cost-effective intervention plans from a subset of possible plans with precomputed effectiveness, and the second greedily composed intervention plans from most cost-effective individual NPIs. The other winning submission—submitted by some of this paper's authors—was the starting point for the approach described here.

In this paper, we describe a framework to help policy-makers design reasonable intervention strategies by dynamically adjusting NPIs. The framework is comprised of three components: a predictor based on the SEIRD epidemiological model that predicts infection trends, a compilation of SECs of NPIs, as found in the literature, and a prescriptor that finds diverse optimized intervention plans. The main methodological novelty of the predictor is that the key parameters of the SEIRD model can be dynamically adapted to any set of given NPIs using a machine-learning model. Intuitively, the machine-learning model decreases the disease transmission rate in the SEIRD model when strict NPIs are in place, and vice versa. In contrast to most related work, our prescriptor uses multi-objective optimization and does not combine the objectives into a single function. As such, it can find near-optimal trade-offs between the costs (SEC) and benefits (reduced number of infections) of NPIs, and presents the results in the form of a Pareto-front approximation. Ideally, the obtained Pareto-front approximation ranges from costly intervention plans, which significantly decrease infections, to cheap but not as effective ones—presenting a set of plans for the policy-maker to choose from. Our methodology was extensively tested: the predictor was tested on data from 194 territories and the prescriptor on data from 50 territories. It yields semantically sensible results, achieves similar or better prediction accuracy than previously proposed models, and furthermore, proposes better plans—at least based on our simulations—than those actually implemented by policy-makers in the studied period (March 2020 to April 2021).

## 2. Methods

We defined an intervention plan as a prescription of which NPIs, and with what strictness, are to be used on each day in a time period. For this study we considered 12 NPIs listed in Table 1, and we denoted this set as *OxNPIs* as it is derived from Oxford's OxCGRT dataset (12) introduced in the following subsection. The task of finding good intervention plans could then be framed as a multi-objective optimization problem—trying to minimize both the number of infections and the SEC that would result from a given plan.

**Table 1:** Social and economic costs for OxNPIs.

**OxNPI**	**Economic**	**Social**	**Combined**
C1: School closing	3.9	11	0.55
C2: Workplace closing	22.0	11	0.96
C3: Cancel public events	1.4	7	0.32
C4: Restrictions on gatherings	1.4	10	0.45
C5: Close public transport	0.3	2	0.09
C6: Stay at home requirements	5.2	12	0.62
C7: Restrictions on internal movement	7.8	10	0.59
C8: International travel controls	6.6	2	0.20
H1: Public information campaigns	0.0026	1	0.04
H2: Testing policy	0.6	1	0.05
H3: Contact tracing	0.1	1	0.04
H6: Facial coverings	0.03	5	0.21

Economic costs are shown as % of GDP loss in the period the NPI was implemented. The social costs are based on domain knowledge and expressed on a 1–12 scale. The combined column is the average of the two costs, when both are normalized to the [0, 1] range.

Given this formulation, we had to solve the following three problems: 1) how to estimate the number of infections in a specific territory, given an intervention plan; 2) how to estimate the SEC of an intervention plan; and 3) how to use both these estimators and a multi-objective optimization algorithm to find different intervention plans. We start by describing the dataset used and then our solution to each of the listed problems in the following subsections.

### 2.1. Dataset

The NPIs used in this study were derived from the “COVID-19 Government response tracker” database, collected by Blavatnik School of Government at Oxford University (12). This database defines the periods in which different NPIs (e.g., “C1: School closing” and “C8: International travel controls”) were implemented in each territory (entities such as countries, US states, or counties of the UK). It also defines their “strictness” in the form of numbers usually ranging from 0 to 3 or 4, which can represent, for example, if all or only some schools were closed. From the NPI list available in the Oxford database, we selected 12 for analysis in this study: H1, H2, H4, H6, C1-C8 (OxNPIs). Their description and the reasoning for their selection can be found in the Supplementary material—Non-Pharmaceutical Interventions.

The number of infections and deaths (note that we are working with “reported cases” which is only an approximation for the actual number of infections) was queried from the same database for the period between March 1, 2020, and April 14, 2021. This database contained 235 territories, of which different subsets were used in different stages of our methodology. For fitting the epidemiological model, all 235 territories were used. Then, some territories were excluded as their data could not be accurately fit with an epidemiological model (e.g., if the number of reported infections were too low or data was missing). This resulted in 194 territories on which we evaluated the predictive model. For each of them, we chose fifty 70-day time intervals, thus generating 9,700 test cases for the task.

In addition to the already described OxNPIs and infection numbers, the following attributes were used to train the machine-learning models: vaccination (13) (one shot, two shots), strains (14, 15) of concern and interest as defined by the World Health Organization (16) testing rate (17), number of hospitalized patients (18), number of patients in intensive care (18), mask use (19), mobility (20, 21), weather (22), holidays (19), and 93 static features characterizing countries and regions (e.g., development, culture, and health) from our previous study (23). “Duration” features were also constructed to capture how long each NPI had been active to date and how much time had elapsed since the first recorded infection case.

Finally, for the prescriptor evaluation, we chose a representative sample of 50 different 60-day intervals. This sample was selected by first defining the “category” for each time interval: the categories were created based on the size of the territory (small/large) and the derivative of the number of infections (slope). The slopes were either constant, moderately steep (falling/raising), or very steep (falling/raising). Altogether, we had 10 categories, and we randomly selected five time-intervals from each. An additional condition for an interval to be selected was to have at least 0.5 average number of daily new infections per 100k of population.

### 2.2. Hybrid machine-learning epidemiological model

To predict the future number of infections we used an epidemiological model that can model the course of the disease given some parameters (infection rate, incubation period, mortality) in combination with a machine-learning model that can estimate these parameters from the active NPIs.

#### 2.2.1. Epidemiological model

We used the SEIRD (24) model, which originates from the SIR family of standard epidemiological models used to study the dynamics of infectious diseases. Even if the SEIRD model is more complex than the basic SIR or SIRD models, it has proven to be more numerically stable than the other two for our purpose, and in addition, the numbers for all five categories were available. The model consists of a set of differential equations (Equation 1). Letters represent the size of a given compartment (*S*usceptible, *E*xposed, *I*nfected, *R*ecovered, and *D*eceased), N is the sum of all compartments, *β* is the infection rate, *σ* is the incubation period (1/days), γ is the recovery rate, *μ* is the mortality rate, and *t* is time. The reproduction number can be estimated as $\frac{\beta }{\sigma }$.


(1) $$\begin{array}{ll} \begin{array}{l} \frac{dS}{dt}=-\beta \frac{SI}{N}\\ \frac{dE}{dt}=\beta \frac{SI}{N}-\sigma E\\ \frac{dI}{dt}=\sigma E-(\gamma +\mu )I \end{array} & \;\begin{array}{l} \frac{dR}{dt}=\gamma I\\ \frac{dD}{dt}=\mu I \end{array} \end{array}$$

In a standard SEIRD model, the parameters *β*, *μ*, and *σ* are constant. In reality—especially in the case of COVID-19—they are highly dependent on various factors, including the NPIs. In related work, there were several attempts at modeling *β* as a function of interventions. In the DELPHI model developed by COVID Analytics (25), the effect of interventions was modeled using an arctan function (26). Zou et al. (27) used machine learning to learn the epidemiological model parameter values from the number of infected and removed (deceased and recovered) cases at time *t*. In our model, we used machine-learning models that used several different features to achieve this task—allowing us a greater flexibility in dynamically changing the parameters, as opposed to what could be achieved with other methods from related work.

#### 2.2.2. Predicting the model parameters with machine learning

The first step of the process was to fit the *β*, *μ*, and *σ* parameters to different territory/time intervals. This was done by finding parameter values that minimize the least squares error in predicting the reported number of infections and deaths. The time series of data for each of the 235 territories were split into intervals based on two criteria: *NPI change* (at least two NPIs change on the same day) and *infection trend* (a 7-day moving-average number of infections that was previously raising, starts falling—or vice versa), and each was fitted separately.

These fitted values were then used as prediction targets for three machine-learning regression models (one model per parameter). When trained, these models would be used to predict the parameters when evaluating different NPIs by the prescriptor.

For the prediction of each parameter, we used the features described in the Dataset section, and some of their subsets. We performed an initial feature selection on the available dataset by employing Recursive Feature Elimination (RFE) with a 10-fold cross-validation. We evaluated both 1) straightforward feature selection (i.e., running the algorithm on all available features), and 2) including the OxNPIs in the selected features and running the RFE only on the remaining features. However, the results showed no significant improvement after the RFE algorithm. For the sake of model interpretability, we selected the features presenting the strongest correlation with the reported number of infections, and ended up with OxNPIs, duration features, historical infections, COVID-19 strains, and vaccination features.

We tested linear regression (28), ridge regression (28), decision tree (28), LGBM (29), XGB (30), CatBoost (31), Elastic Net (28), Bayesian ridge (28), SVR (28), and Random Forest models (28). The models were compared with 10-fold cross-validation where the train/test splits were performed territory-wise, meaning that all instances of a territory were in either the test or the train set. Keeping all instances of one territory in the same set was important since consecutive instances were typically similar.

In the cases of linear and ridge regression, the regression coefficients for the final model were calculated as the mean values of the coefficients generated in the 10-fold cross-validation. The “H1: Public information campaigns” regression coefficient initially had an excessive value because the corresponding NPI was essentially always present (and was thus used by the model almost as the intercept). We, therefore, manually adjusted it based on Haug et al.'s (32) study. Specifically, we used the four NPIs for which there was a good match between our categorization and the one presented by Haug et al.: “C1: School closing,” “C7: Restrictions on internal movement,” “C3: Cancel public events,” and “C5: Close public transport.” We computed the ratio between the decrease in reproduction rate (*β*/γ) for these four NPIs (32), and the decrease for “H1: Public information campaigns.” We then multiplied our coefficients for the same NPIs with these ratios, which yielded four possible values for the H1 coefficient. We used the average of these. We then re-ran the regression with fixed relations between the NPI coefficients, so that the relation between them and other coefficients could be readjusted.

Since the parameter *β* (infection rate) was most strongly affected by NPIs, and since we are aware of no strong reason why the other two should be, we also considered predicting *β* only. And since the parameters of the model are not independent, we considered using some as features for the prediction of others. However, both of these approaches gave worse results.

#### 2.2.3. Prediction pipeline

The goal of the prediction pipeline is to predict the number of infections given an intervention plan (which OxNPIs are used on a given day). To do so, we create a feature vector by joining the OxNPI data with the remaining features. Then, for each day, a prediction of all three parameters is made with the three respective machine-learning models.

Next, for the time interval leading to (but not including) the prediction interval, the fitted parameters are queried. We assume that the parameters at the beginning of the prediction interval should be the same as the fitted parameters at the end of the last one directly preceding it. Thus, the machine-learning predictions are normalized as *β*_*i*_ = *β*_last_/*β*_0_, where *β*_*i*_ is the value of the predicted parameter *β* on the *i*-th day, and *β*_last_ is the last known fitted value of *β* preceding the prediction interval. Parameters *σ* and *μ* are normalized similarly.

If the parameters for any day are such that the reproduction rate exceeds five, then the value of *β* is reduced until the reproduction rate falls to this threshold value. This is done because such high reproduction rates do not appear in real-life data, but they might be predicted due to some edge case in machine learning. All parameters are smoothed using weighted decay (α = 0.2), as we assume that all parameters are changing smoothly.

When the parameters are estimated for each day, they are inserted into the SEIRD model, which can then produce the number of infections for each day. The starting value of the “Exposed” compartment is set in a way such that the predicted and reported numbers of infections match on day zero.

### 2.3. Socio-economic costs of different NPIs

The collection of socio-economic costs (SEC) of individual OxNPIs was not the primary focus of our work, but nonetheless we compiled a sensible set in order to properly test our methodology. The collected SECs were derived from a set of costs from related work and from the opinion of a domain expert. Due to the available literature, the costs are likely to contain a bias toward Western countries, and most data is based on reports and gray literature.

In the study, we used the values listed in Table 1, but the methodology is rather general and a policy-maker can easily adapt it to produce a set of SECs for a specific territory—possibly also implicitly expressing their preferences on what NPIs to avoid (by assigning them higher costs). The combined SEC is made simply by normalizing both costs to the [0, 1] range and then averaging both. While this number does not have a good interpretation, it does rank the OxNPIs according to their SECs. The costs are given for the case in which the NPI is implemented with its maximum strictness. For other strictness levels, the costs were linearly scaled down (in rare cases, a custom social cost was defined and used instead of the linearly scaled value). In addition, the “C6: Stay at home requirements” NPI requires the implementation of the C1, C2, C3, C4, C5, and C8 NPIs. Thus, even if it did not have the highest cost, the overall cost implicitly includes the costs of all other listed NPIs.

#### 2.3.1. GDP loss

Because the available findings on economic cost of NPIs differ in terms of the setting and time, they were normalized to represent the % of GDP loss caused by the NPI while it was in effect. Country-specific GDP values (US $) were used (33). For example, if the “C3: Cancel public events” NPI is active for 1 month and it has the cost of 1.4, then our method assumes that the GDP in this month is 1.4% lower than usual—note that this is not the annual GDP loss but that for the predicted period. The complete overview of the cost data used can be found in the Supplementary material—GDP cost. While there is some overlap between the NPIs, we have explicitly modeled this only in case of C6 as previously described.

#### 2.3.2. Social impact

While economic costs were available for most OxNPIs, the literature on social costs was far more scarce. We thus placed the ranking of OxNPIs by social costs on a theoretical foundation, but we could not justify the numerical costs as solidly. In addition, according to the literature, these costs may vary across countries (e.g., collectivistic vs. individualistic countries); however, we applied standard levels for all WEIRD countries (i.e., for Western, Educated, Industrialized, Rich, and Democratic, a common grouping in psychological studies). To estimate the social costs, we ranked the OxNPIs from the highest to the lowest based on the perceived strain, dread and loss, perceptions of restricted freedoms, and constraining behaviors (i.e., on the negative impact of each measure on behavior, attitudes, and one's well-being). Using the rational choice theory, we assumed that the higher the perception of strain, dread and loss, the more negative is the impact and the higher are the social costs. Understanding human behavior and risk perception is central to effective pandemic management, and thus we applied insights from social and behavioral sciences to inform our assumptions on social impact. For determining the cost of individual policies see Supplementary material—Social cost discussion.

### 2.4. Proposing interventions

The task of proposing intervention plans can be mathematically formulated as a multi-objective optimization problem with two objectives that need to be minimized: the total number of infections (*f*_1_) and the SECs of the proposed plan (*f*_2_). The two objectives are conflicting since an effective way to slow down the spread of infection requires a stringent intervention plan with expensive NPIs. The first objective is expressed as the total number of infections predicted from the HMLE model, while the second objective is the cost of NPIs averaged over the plan's duration. The problem is constrained by limiting the number of new daily infections to 150 per 100k residents. This is done as the plans with more infections were not considered useful to policy-makers and almost never appear (< 1%) in real-life data in the studied period.

**Figure 1:**
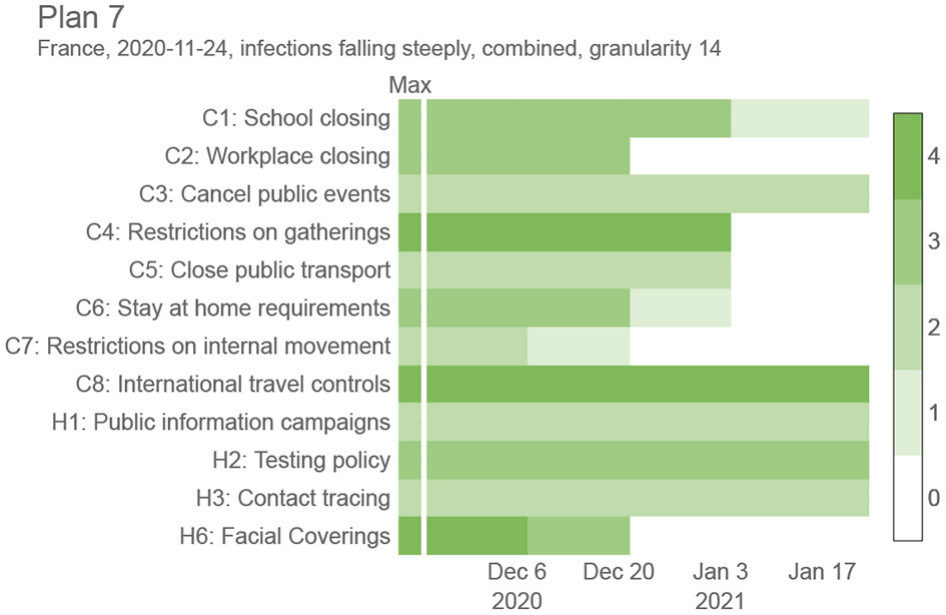
Sample intervention plan for France between November 24, 2020, and January 24, 2021, with a granularity value of 14 days. Refer to Figure 6 to see how this plan compares against other proposed plans in the same period.

The proposed intervention plans are composed of OxNPIs that can vary over time, but are restricted to last at least *g* days in a row, where *g* is a predetermined parameter we refer to as *granularity*. An NPI, for example, “C2: Workplace closing,” can be applied with different levels of strictness (0—no policy, 1— closure recommended, 2—closure for specific sectors, 3—closure for all-but-essential workplaces). With this in mind we can formally define the intervention plan—a solution to the proposed optimization problem—as a 12 × *n* integer-valued matrix, *P*, where its 12 rows correspond to the 12 OxNPIs and *n* is the number of time slots determined by the given granularity and the whole period (e.g., Figure 1 contains *n* = 4 time slots resulting from a granularity value of 14 days and an interval length of 60 days). In detail, *P*_*ij*_ indicates the strictness of the *i*-th NPI in the *j*-th time slot. In particular, we tested five values for granularity: 1, 3, 7, 14, and 30.

Based on the multi-objective formulation of the proposed optimization problem, the experimental evaluation aimed at finding sets of trade-off intervention plans representing approximations for Pareto fronts. For this purpose, we used the Nondominated Sorting Genetic Algorithm II (NSGA-II) (34) equipped with a Constrained Dominance Principle (CDP) (34) to handle the constraint. NSGA-II belongs to the group of evolutionary algorithms, and as such, it imitates the biological evolution to search the space of possible intervention plans and find plans with good trade-offs between the two objectives.

The optimization problem was solved using two NSGA-II internal solution representations: the full representation defined by the matrix *P* and the condensed representation defined by a vector of length *n* where the *j*-th variable corresponds to the maximum SEC allowed at the *j*-th time slot. The second representation was considered due to the significant reduction in the search space dimensionality (from 12*n* to *n*), allowing for much faster convergence than the high-dimensional search space for the full representation. While the full representation can be used without modifications, the condensed representation needs to be decoded to the intervention plan before evaluation. This is achieved by replacing the SECs with OxNPI values. The OxNPI combination to replace each SEC is selected as the one with the lowest projected infections out of those within the allowed SECs. This mapping is computed in advance, by having all OxNPIs combinations sorted based on their effectiveness (by using linear model's coefficients for each NPI), so that the most effective combination that does not exceed the cost threshold can easily be selected.

The one-point crossover was used as the crossover operator and the random resetting as the mutation operator. Additionally, the crossover probability was set to 0.9 and the mutation probability to 1/*D*, where *D* equals 12*n* for the full representation and *n* for the condensed representation.

## 3. Results

### 3.1. Predicting infections

**Figure 2:**
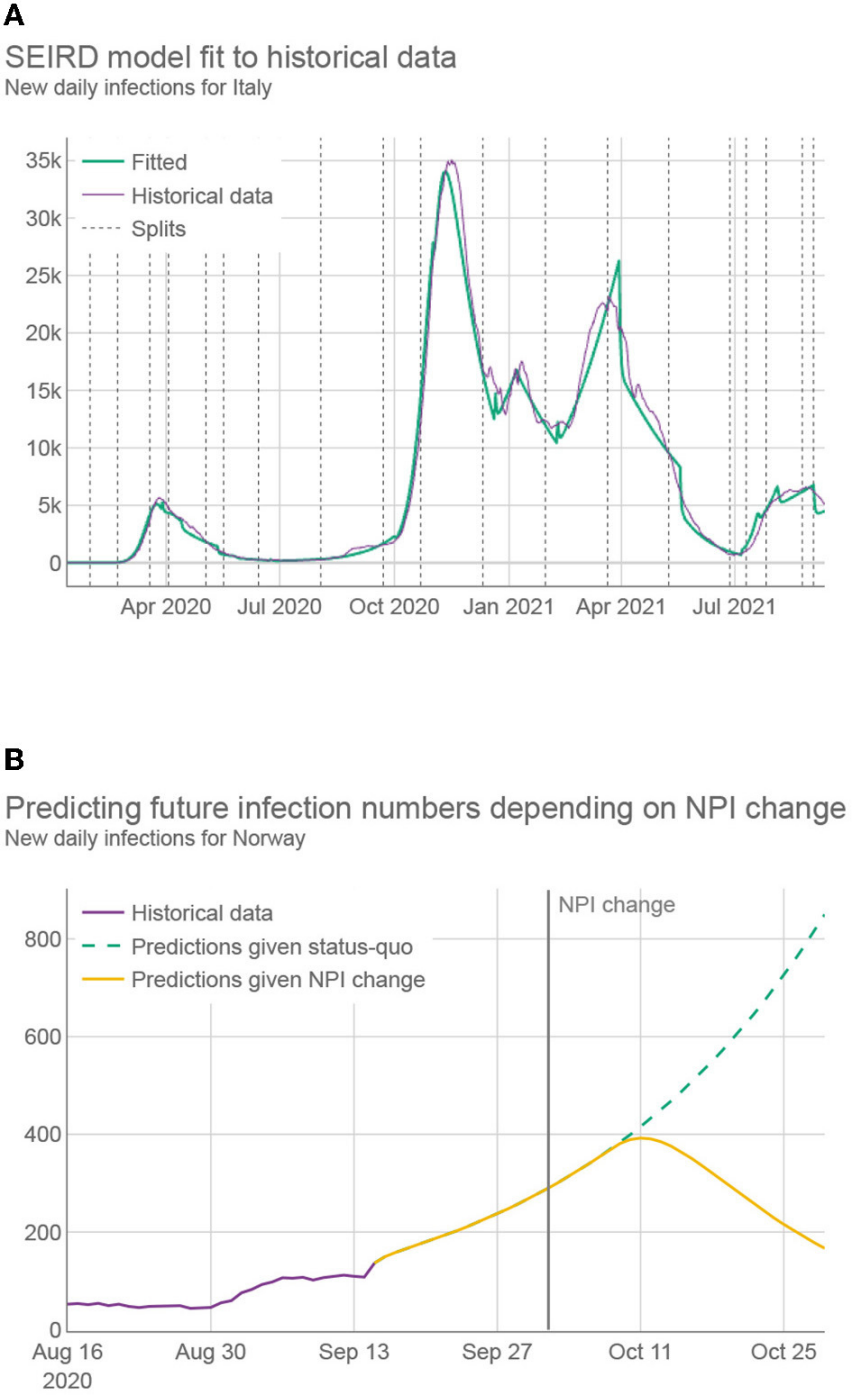
**(A)** Daily infections for Italy over 20 months (purple) together with the predictions using best fitted SEIRD model (green). Fitting was conducted by first splitting the data into segments, represented by dashed vertical lines, where at least two NPIs were changed with respect to the previous segment. Different segments use different fitted parameters. **(B)** Daily infection predictions for Norway, made both by using only fitted parameters (green) and by using parameters adapted by machine learning, which reflect the change to more strict NPIs (yellow).

While the SEIRD model on its own is accurate in predicting the future in cases where NPIs are not changing and historically-fitted parameters can be used (see Figure 2 and Supplementary material—Estimating the prediction error), it does not correctly predict the infection trends following a change of the NPIs— which is essential if the framework is to propose which NPIs to use in the future. Ideally, as the NPIs change, the parameters of the SEIRD system would be adjusted accordingly, taking into account their changed impact on the disease transmission rate. An example of such behavior can be seen in Figure 2, as generated by our HMLE method.

**Figure 3:**
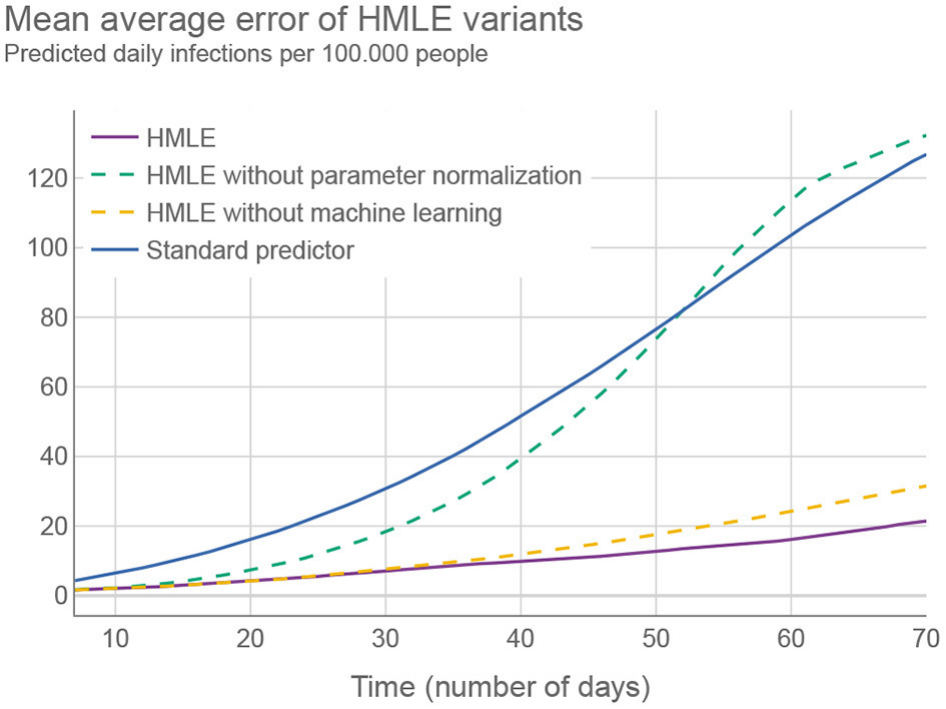
Different versions of the HMLE method compared to the “standard predictor” (9). Testing was conducted on 50 random time intervals for each of the selected 194 territories.

To assess the performance of the HMLE method, we show in Figure 3 that our predictor significantly outperforms the “standard predictor” provided by Cognizant in the second phase of the XPRIZE competition (9) (for details of this test, see Supplementary material—Estimating the prediction error). This is a predictor published prior to the competition (10), which represented the state of the art for NPI-dependent prediction at the time. The mean average error (MAE) of our predictor is 5.9 times lower on day 70. To explore what contributes to the increased performance, we compared the full implementation to two additional versions of our method: 1) one that relies only on machine learning to set the parameter values of the SEIRD model without normalizing them using the last known fitted parameter values, and 2) one that retains the last known fitted parameter values throughout the forecast period, without using machine learning to account for NPI changes. The experimental results showed that the parameters predicted by the machine-learning model are less appropriate on average, than the last known fitted parameters; when normalized, however, they outperform the last known fitted parameters. The benefit of machine learning does not appear to be huge, but it is significant in case of important NPI changes, as demonstrated in Figure 2.

**Figure 4:**
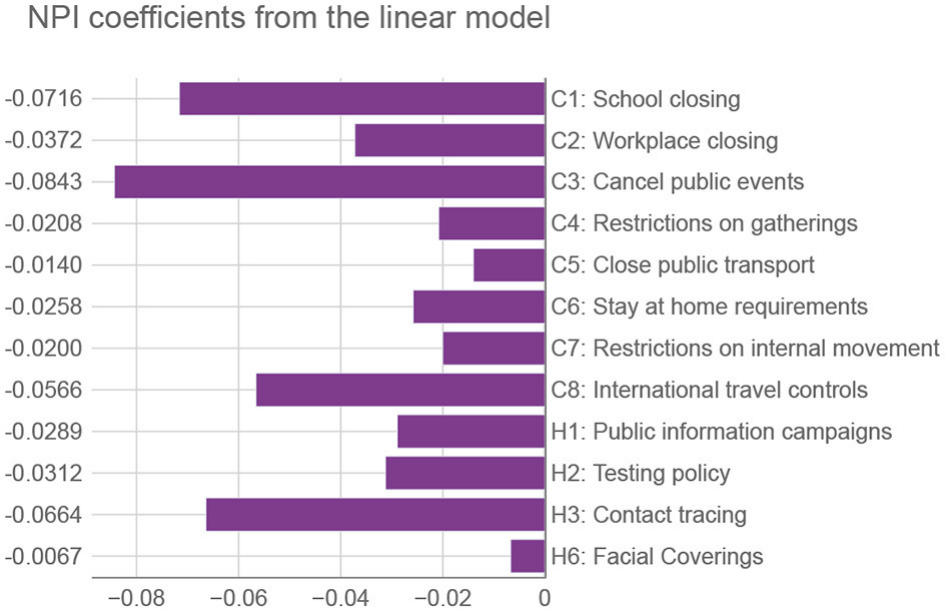
Coefficients from the linear model corresponding to OxNPIs. We use the terminology of Oxford's COVID-19 Government Response Tracker (12), with containment (C) and health (H) categories. Relative values of NPIs can signify their importance for reducing the number of infections—the larger the negative value, the more they suppress the infection spread.

Of all machine-learning algorithms tested (see Supplementary material—Estimating the prediction error), the Ridge classifier (a type of linear model) had the highest accuracy. Aside from the prediction accuracy, the model has an additional advantage—it is easily interpretable. Figure 4 lists the coefficients corresponding to the normalized OxNPI strictness values. Given this normalization, the model's coefficient magnitude can indicate relative NPI importance. Our model's most important intervention is the cancellation of public events, which is consistent with the related work that typically ranks it among the top NPIs (32). Next is school closure, which additionally results in some parents staying at home, so its importance is not surprising. These two are followed by contact tracing—which is difficult to execute well, and other sources do not rate this NPI as high. In the fourth place are international travel controls, which played a big role in some countries, particularly in the early stages of the pandemic. The importance of this NPI was corroborated by Haug et al. (32). Other NPIs have notably lower coefficient values. This may come as a surprise for “C2: Workplace closing,” “C4: Restrictions on gatherings,” and “C6: Stay at home requirements,” but it should be noted that 1) these three NPIs have a large overlap with each other and with other NPIs, and 2) they were usually instituted when the epidemiological situation was grave, with many NPIs in force simultaneously, thus making it very difficult to properly isolate the importance of each of them. This is why in these cases the assigned regression coefficient do not necessarily correctly reflect their relative importance. Nonetheless, their sum is close to the largest single coefficient. Of note, the NPI features were not the only ones included in the model, but the coefficient values of the others were an order of magnitude lower than those listed here.

Finally, for a direct comparison with related work, the HMLE model described here is an improved version of the one used in the XPRIZE challenge, which was ranking between the 1st and 4th place during the 2 month prediction period on real data for 235 territories (35).

### 3.2. Proposing interventions

Figure 1 shows a sample trade-off intervention plan consisting of NPIs changing in time (*g* = 14), to provide a better intuition for the end goal of this work. It lists all 12 OxNPIs, their maximum value, and some sample values. For example, the intervention plan depicted in Figure 1 suggests to close all-but-essential workplaces from November 24, 2020, to December 20, 2020, but relaxes most countermeasures after that.

The experimental setup was established based on some initial experiments. NSGA-II was run with a population of 100 solutions for 500 generations (50k plan evaluations in total). This number of evaluations proved to be sufficient for convergence using coarser granularity values. Moreover, increasing function evaluations did not significantly improve the results, even for finer granularity values. For this reason, 50k evaluations represented a good trade-off between the framework's effectiveness and efficiency.

We tried to identify the best value for granularity and we compared five values: 1, 3, 7, 14, and 30. Theoretically, with a finer granularity, we can achieve at least as good intervention plans as with a coarser granularity. However, with finer granularity, aside from being impractical in real-life use, the search space of the optimization problem increases significantly, and the optimization cannot always find the best solutions. Then, we compared the two ways of representing intervention plans during optimization: full vs. condensed.

In all experiments in this section, the optimization was tested on 50 representative territory/time interval examples (see Section 2.1.). Due to the stochastic nature of the employed optimization approach, the presented results were obtained after running the optimization 31 times on each example, as this is enough to obtain statistically relevant results. To measure the effectiveness of the multi-objective optimization, we used the well-known hypervolume indicator (36)—the volume of the area bounded by the Pareto front approximation and a user-defined reference point. The medians of the obtained hypervolumes were used for testing the statistical significance of one granularity/representation being better than the other.

We first compared different granularity values when using the condensed representation. According to the Friedman test, we observed statistically significant differences between granularity values: χ^2^(3) ≈ 150.678 and *p* < 0.01 for social weights, χ^2^(3) ≈ 119.309 and *p* < 0.01 for GDP weights, and χ^2^(3)≈106.139 and *p* < 0.01 for combined weights. *Post-hoc* analysis with Wilcoxon signed-rank test and Holm's correction to adjust the *p*-values indicated that the granularity of 14 days was the most effective among the tested values (see Supplementary material—Details about the multi-objective optimization results).

**Figure 5:**
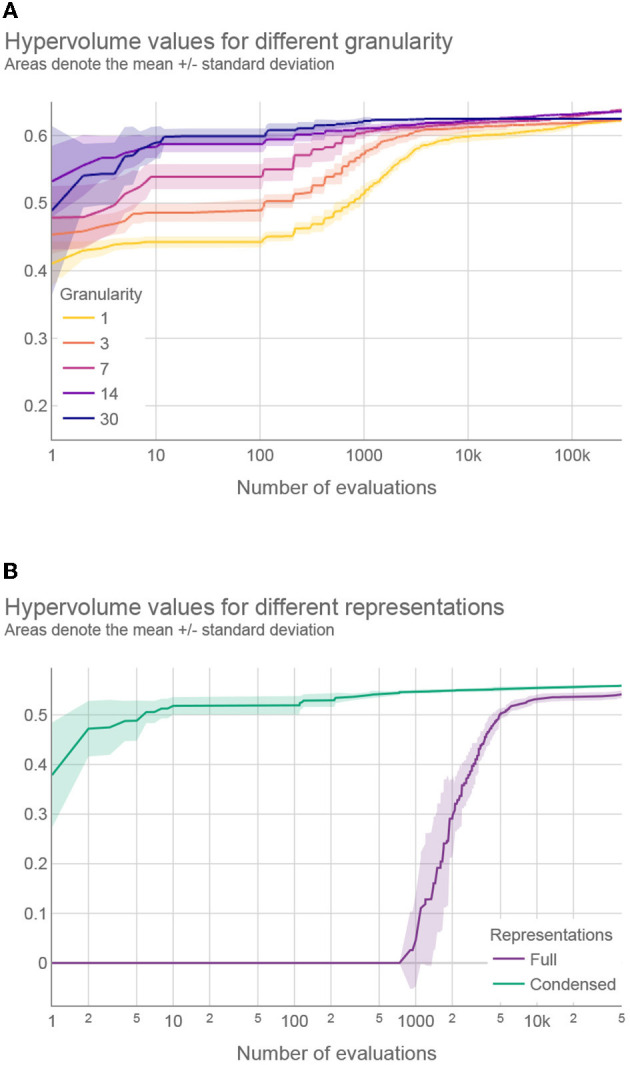
**(A)** Hypervolume progress for different granularity values using condensed representation and **(B)** hypervolume progress for full and condensed representations with the best performing granularity values. A logarithmic scale is used for the horizontal axis (number of evaluations).

Our results confirm that the optimization algorithm struggles to find near-optimal interventions plans with fine granularity values, due to the increase in search space dimensionality. For example, Figure 5 shows the hypervolume progress—the improvement of the results during the optimization—averaged over 31 optimization runs where the number of intervention plan evaluations was experimentally increased from the default 50 to 300 k. This was done to estimate the optimization behavior and convergence when using a large number of evaluations. As we can see, although the results obtained with a granularity value of 7 days eventually surpassed those results obtained with a granularity value of 14 days (at around 230 k evaluations), the computational time required to obtain better results using finer granularity values was almost five times longer, and the gain in the solutions' quality was negligible compared to the additional computational resources spent (Figure 5). In addition, the extremely small differences between the granularity value of 7 or 14 days are practically irrelevant since, in a real-world scenario, the objectives cannot be measured and predicted with such accuracy. Moreover, it is easier to implement intervention plans that change with coarse granularity values (37); therefore, a granularity value of 14 days seems to be a reasonable choice.

A similar investigation was devoted to finding the best granularity value for the full representation. The results of the statistical analysis revealed significant differences in hypervolume values and showed that the granularity of 30 days is the best performing value for this representation. The complete results can be found in the Supplementary material.

Finally, we compared the full and condensed representations with the best performing granularity values. According to the Wilcoxon signed-rank test, the condensed representation outperformed the full representation for all types of weights (*p* < 0.01). Moreover, Figure 5 compares the hypervolume progress between the two representations on a typical problem instance, where a much faster convergence can be observed with the condensed representation. This was not unexpected since the applied optimization approach performs significantly faster for low-dimensional search spaces. The results provided in the following sections were obtained using the condensed representation with a granularity of 14 days since this was the best performing setting.

### 3.3. Intervention plan interpretation

**Figure 6:**
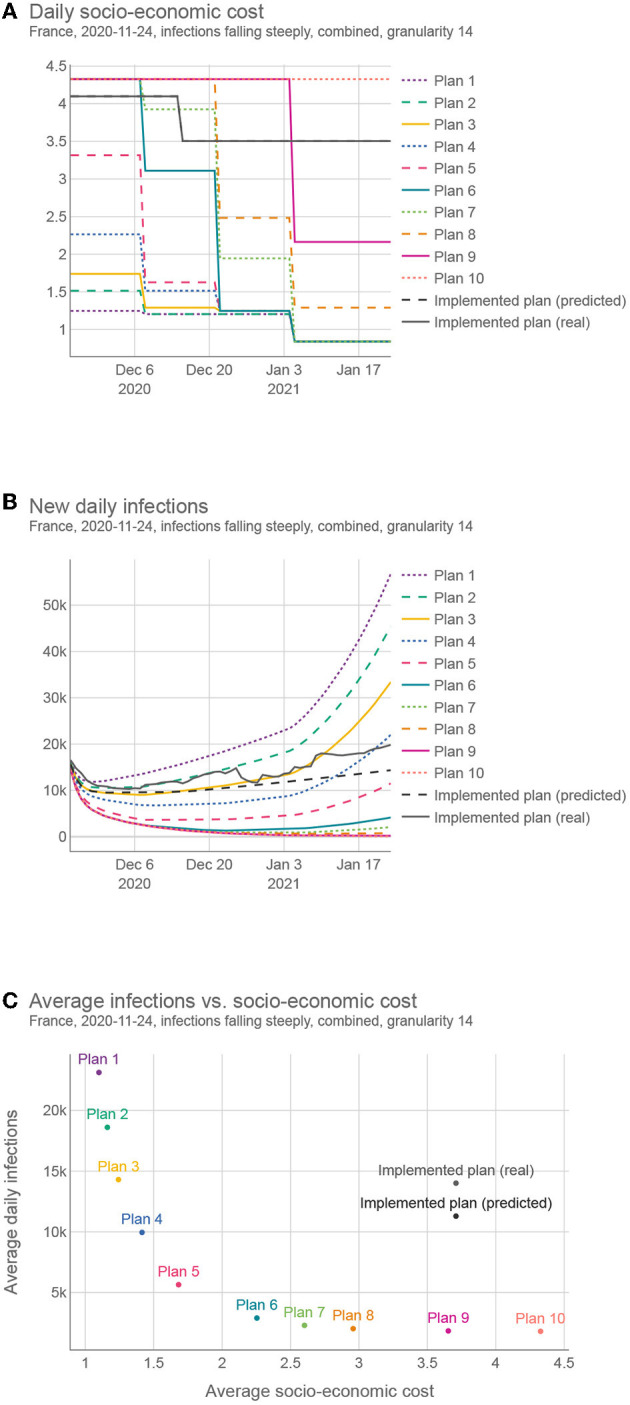
Comparing different intervention plans for France. **(A)** Shows the SEC (GDP loss + social cost) over time. **(B)** Shows the predicted number of infections, **(C)** Shows the trade-offs between the two criteria (SEC and the number of infections) for different plans.

To better understand how different intervention plans compare, we generated 10 different intervention plans for the same territory/time interval as that shown in Figure 1 (among all intervention plans obtained by the optimization, we selected the 10 that are the furthest from each other in the objective space). Figure 6 shows for each plan 1) the strictness of the interventions over time, 2) the resulting infection curve, and 3) the comparison of the 10 plans in terms of the number of infections and strictness. This example was done with the granularity of 14 days using the “combined” cost for the interventions. However, we generated plans using all different intervention costs and both 7 and 14 granularities for the same 50 test cases that were used for testing multi-objective optimization. This complete set of results can be found on the results webpage (38). For a subset of these results, see Supplementary material—Sample intervention plans.

The proposed plans present a wide range of trade-offs between the two objectives, and policy-makers can choose the one most suited to their needs. In addition, they can change a portion of the plan if deemed necessary and evaluate it again. This whole framework is available as a web tool (39), currently implemented for Slovenia.

The proposed solutions were compared with the real-life solution implemented in the same territory/time. This real-life solution was estimated in two ways, *(real)* using the actual reported number of infections and *(predicted)* using the predicted number of infections given the implemented NPIs. As the real SEC was, in most cases, unknown, we used the same estimation function for the *real* case as for the proposed plans. In all 50 test cases, the proposed solutions compared favorably against the *predicted* case, and in 47 test cases, the proposed solutions compared favorably against the *real* case. On average, we could find a solution with the same number of infections but with 47.1% lower SEC, or a solution with the same SEC but 68.8% lower number of infections (for details, see Supplementary material—Comparison of the proposed and implemented solutions).

**Figure 7:**
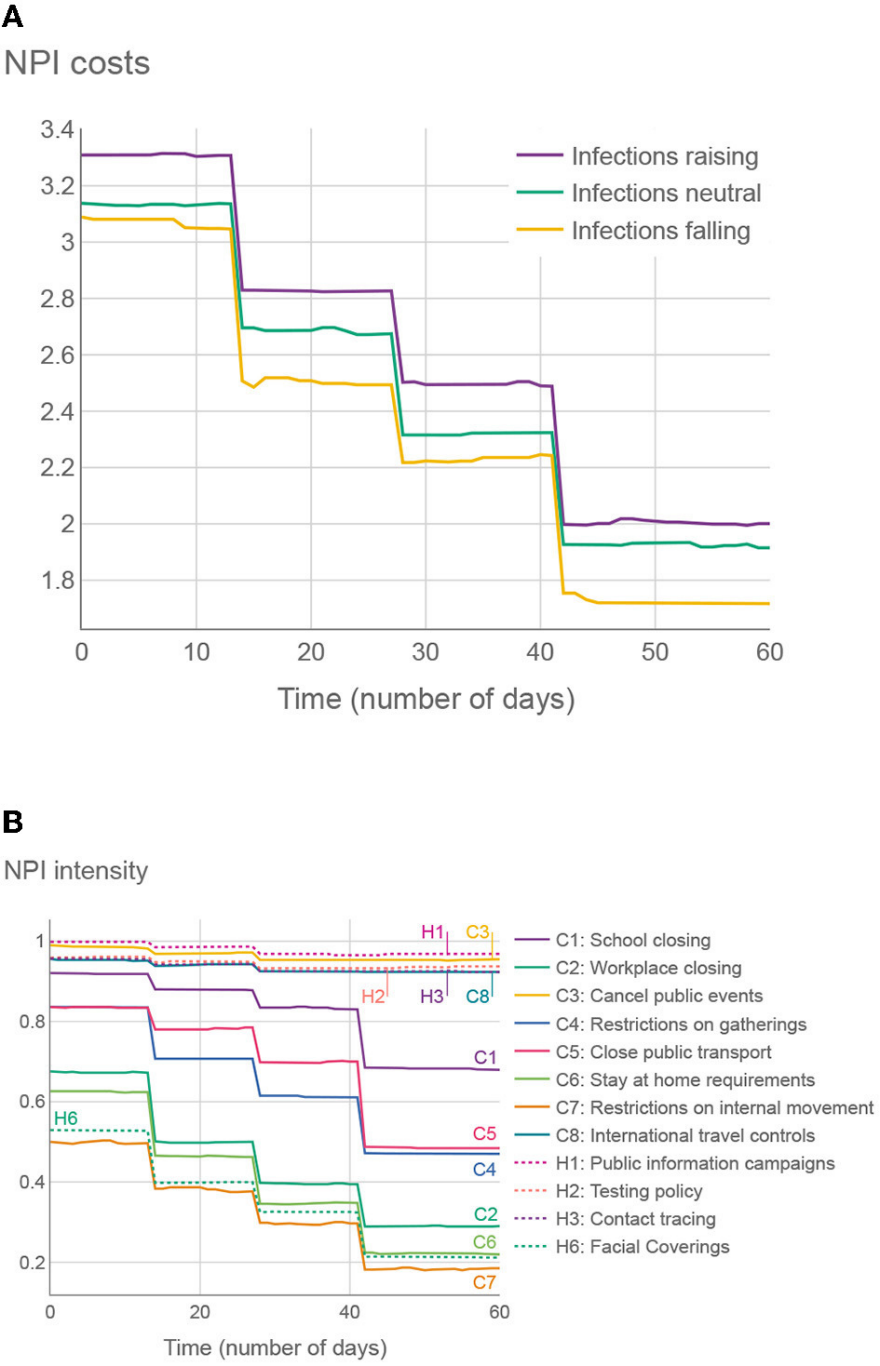
**(A)** Average SEC across all 500 proposed intervention plans (50 test cases, 10 plans on each), given the number of days since the intervention has started. **(B)** Average OxNPI strictness (normalized to 0-1 range) across all 500 proposed intervention plans, given the number of days since the intervention has started.

To explore the trends in the structure of the intervention plans, we considered two experiments. First, we averaged the OxNPIs costs across all plans in all test examples, aggregated on a daily basis. The results in Figure 7 show that, on average, the intervention plans are the strictest at the beginning and then gradually become more relaxed. It also shows that in test intervals where the infections were falling, the overall strictness is lower than in cases where infections were raising. The difference might not be as big as expected, again due to the optimizer providing a wide range of intervention plans.

In the second experiment in Figure 7, we show the average strictness of individual OxNPIs, again averaged across all intervention plans in all test cases. The NPIs with high average intensities can be considered to provide good trade-offs between their cost and effect.

The structure of the proposed plans was generally quite consistent from one territory to another. One can reason that—since the NPIs tend to have similar cost and benefit (at least in relative terms) regardless of the current epidemiological picture, and the prescriptor is designed to create solutions with a wide range of costs—the resulting plans will, in most cases, share a common structure that will be somewhat adjusted for different territories/time intervals. Another way of looking at it is to consider that reducing the number of infections when there are, for example, 1,000 daily infections has the same importance to the algorithm as reducing the number when there are 3,000 daily infections. It is up to the policy-maker to consider when the situation merits selecting a different proposed intervention plan with a lower/higher SEC.

## 4. Conclusion

The presented framework can generate efficient intervention plans to fight a pandemic, and can evaluate their effect and costs. This can greatly help policy-makers to pursue sensible intervention strategies and reason about their strengths and weaknesses. We showed that intervention plans it generates—at least when evaluated with our methodology—are better than past interventions generated by policy-makers. Since very few NPIs are still used against COVID-19, the main value of our framework is in pandemic preparedness: both as a tool to fight future pandemics (for which it would probably not require many modifications), and as a demonstration of the value of artificial intelligence in this area in general. All data used to generate the figures is available in our repository (38). The same repository also contains all final results. All code used in the production of the results is available in our code repository (40).

### 4.1. Intervention plan insights

In general, the most effective NPIs were school closing, canceling of public events, workplace closing, contact tracing, and international travel controls. This list is not surprising as it is similar to the findings in the literature (1, 2). When accounting for cost (which is usually not done), the most efficient NPIs were information campaigns, canceling of public events, and international travel controls, followed by school closing. The least efficient were the restrictions on internal movement, facial coverings, stay-at-home requirements and workplace closing. The latter two are on the list due to their high cost; in particular, the former can usually be substituted with a combination of other more socially acceptable NPIs. The low placement of facial coverings was surprising. Perhaps this is due to masks being somehow inconsistently applied, which may result in bad training data—or alternatively due to "facial coverings" NPI being almost always active, which made it difficult to isolate its effect. Finally, it could be the case that its social cost was overestimated in this study and it should be reduced in potential future analysis.

An additional benefit of the framework, aside from calculating the cost benefit of individual NPIs, is that it can present a timeline of NPI changes that adapts to the current epidemiological situation. In most cases, the approach “start with a strict policy and reduce it over time” seems to be the most effective. We have also shown that adapting the NPI policy every 14 days is enough to get almost ideal cost/benefit as with finer granularities (e.g., adapting every 3 days provides negligible benefits). Intervention plans made and changed on a monthly basis were found still acceptable; however, using a granularity value of 14 proved to be generally more robust. This could be a valuable finding as frequent changes in NPI policy make adherence difficult and can probably increase socio-economic costs (although we did not model this explicitly). For comparison, we analyzed how often were NPIs changing in real-life situations. For 80% of countries, the median time before changing at least one NPI was somewhere between 14 and 30 days and approximately 90% of countries changed their NPIs at least once, under 14 days of the last change.

### 4.2. Technical advantages

The following are the key innovations introduced: 1) combining machine-learning and SEIRD models in a way that allows the SEIRD parameters to be adapted to different NPIs and thus simulate their effect on infections; 2) using historically fitted parameters to normalize the values output by machine learning in order to adapt predictions for each territory; 3) using multi-objective optimization for finding the best intervention plans in combination with a “condensed” solution representation—facilitating a highly efficient search.

We argue our predictor to be state-of-the-art. However, it was designed and trained for the whole world, and it is almost certain that for many specific territories, a better predictor could be/was developed.

Similarly, while the proposed OxNPI costs are carefully considered, they can certainly be improved upon, especially for specific territories. In future work, the whole SEC model can even be made more complex, i.e., non-linearly accounting for the NPI duration. To take all of this in consideration, we made our whole methodology highly modular, so that each part can be substituted by a similar one if necessary—or one can simply adjust the parameter values of the current components.

### 4.3. Limitations

A drawback of the proposed framework is the negligible effect of vaccinations in the models. While we used some vaccination data, the vaccinations were not widespread at the time of data collection. This can be remedied in future work by using more recent data and probably adding another compartment that models vaccinations to the epidemiological model.

Second, the infection predictor can sometimes become unreliable when predicting for two or more months in advance. We thus recommend that it should be mostly used for shorter periods (30–45 days in advance) and then the predictions should be updated in real time as new data become available. The predictor also becomes unreliable when the number of infections is growing very quickly. Due to the nature of exponential growth, even a small misprediction of a parameter of the SEIRD model can quickly lead the model astray. The problem is compounded by people spontaneously behaving more cautiously during severe disease breakouts, which affects the infections but is not recorded in NPI data. This effect is difficult to avoid, so it should be taken into consideration when analyzing the proposed plans. It should also be noted that infection prediction is used as the basis for NPI prescription (it is used to simulate the effects of different intervention plans), and thus any error in the former affects the latter. This effect is also difficult to avoid or even evaluate, as only one intervention plan can be executed at the same time in practice.

Models were made based on the COVID strains active in the studied period and would have to be slightly adjusted in order to be used for the currently emerging or future COVID strains.

Last, we used the reported number of the infections as one of the objectives—and one can argue that some other metric, such as the number of hospitalizations or deaths might be more appropriate. The hospitalizations were rejected in this study as the data needed was available for only 33% of the studied territories, while infections were preferred over deaths to match the Pandemic Response Challenge competition. Nonetheless, effectively the same methodology (with some tweaks to the epidemiological model) could be used to study the other mentioned criteria.

## Data availability statement

The original contributions presented in the study are included in the article/Supplementary material, further inquiries can be directed to the corresponding author.

## Author contributions

VJ, ML, and MG conceived the experiments. NR and DS collected and prepared the dataset. NR, AV, DS, and CD conducted the experiments. SV, DD, and JJ provided analysis of NPI costs. TT provided visualizations. VJ, AG, and ML analyzed the results. All authors reviewed the manuscript. All authors contributed to the article and approved the submitted version.
