# Supplementary material for: Optimizing non-pharmaceutical intervention strategies against COVID-19 using artificial intelligence
Source: Front Public Health. 2023 Feb 13;11:1073581. doi: 10.3389/fpubh.2023.1073581 (PMC9970257; doi:10.3389/fpubh.2023.1073581)
Supplement: Supplementary file 1 [file Data_Sheet_1.PDF]

Supplementary Material

Non-Pharmaceutical Interventions

All NPIs considered in this study are listed in Tables S1 and S2. For each NPI, we list their possible levels of strictness and short description of each one (all information taken from the official website Hale et al. (2020)). We have not considered *Economic* policies and policies H4, H5, H7, and H8 from the same database, as they do not directly affect the number of infections. Similarly, *Vaccine* policies were not considered, as they were largely not yet in effect at the time of this study. This selection matches that made by the Pandemic Response Challenge organizers.

Estimating the prediction error

When the parameters of our SEIRD model are fitted to best match the data, the predictions closely match the actual number of infections. The average of mean absolute error was 1.64 per 100 000 people and the distribution is shown in Fig. S1. This shows that although relatively simple, the SEIRD epidemiological model has an expressive power to accurately model the progress of COVID-19 infections, given "ideal" parameters.

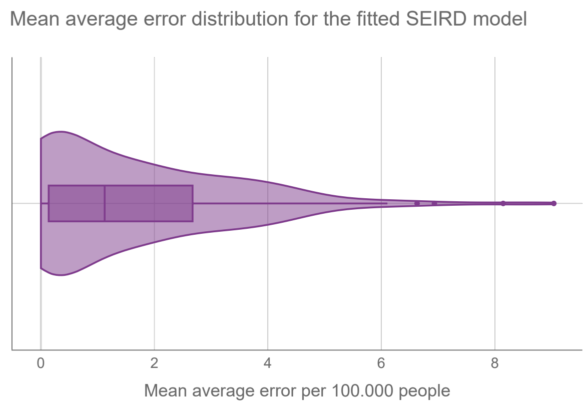

Figure S1. Distribution of the mean average error for the fitted SEIRD model.

Then, we made the prediction of the number of COVID-19 infections using the HMLE method with predicted parameters instead of the fitted ones. All comparisons were made on 50 random time intervals from each of the 194 territories. For each method, we compared the difference between the actual and predicted numbers of infections, for each day of prediction – up to 70 days in advance. Finally, for each day of prediction, the results from all territories were aggregated and the mean average error (MAE) was calculated. Before summing the errors, they were first normalized using each territory's population. The top 5% of errors were removed – this was done as the epidemiological model is exponential in nature and a wrong parameter prediction can, in some cases, result in a numerous infections (e.g., it predicts that the majority of the population will be infected shortly). Such errors would numerically overshadow all others, and were thus removed. Note, however, that even without this error removal, the order of all compared methods remains the same.

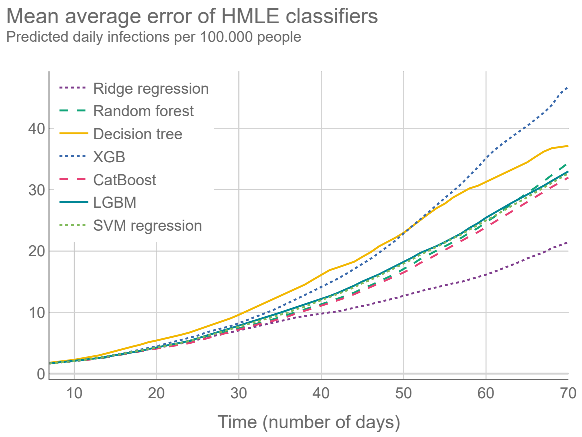

Figure S2. Different classifiers used in the HMLE model.

We compared the performance of using different machine-learning algorithms in our HMLE method. The results in Fig. S2 show that the Ridge linear model yields the smallest error, which is significantly better than the alternatives (MAE is 1.49 times lower on day 70). One explanation for its good performance is that it does not over-fit the data due to its simplicity, while the other is that linear equations seem to describe the parameter changes given different NPIs well. Due to the observed performance, it was used in all other experiments. Similarly, in all cases, the prediction error was increasing with the length of the prediction interval, and thus, prescriptions longer than 2 months were not used for multi-objective optimization.

Social cost discussion

Individuals make decisions by weighing the costs and benefits of NPIs, with emotions driving risk perceptions and making human behavior less predictableBavel et al. (2020). Using this insight, we estimate that stay-at-home requirements (when not sick) impose the highest social costs (cost of 12). Namely, humans are social beings; when introduced over a longer period, lockdown represents the ultimate human challenge. Trumping all other interventions, its harmful effects (on well-being, domestic violence, and society at large) make it the most costly NPI. While we have not modeled this explicitly, it is of note, that people will better comply (cost will be perceived as lower) when lockdown is short and has a fixed start and end date.

Once lockdown is lifted, school and workplace closing, as well as restrictions on internal movement and gatherings, closely follow in cost (11 and 10) because they have similar effects: they limit social relationships, including child's development, and personal and family income (i.e., household purchasing power). There is a time trade-off with these interventions, with longer and more frequent closures and travel restrictions increasing the costs. Moreover, longer workplace closures and travel restrictions are likely to result in the breakdown of general compliance. Hence, for these interventions to be effective, timing and length are key.

By contrast, public information campaigns as key to compliance have the highest social benefit. Elaboration of risks and severity of possible implications motivate cooperative behavior, which is central to managing the pandemic. Thus, the smarter the campaign, the higher the compliance and the benefit. Similar underlying logic applies to the test and trace policy; targeting high-risk individuals, it is an essential component of pandemic management. The cost of these NPIs is thus the lowest (1).

We arranged the costs of the remaining NPIs between these extremes. Cancelling of public events is a milder version of restrictions on gatherings (7). Closing of public transport was typically in force together with other NPIs, which reduced the need for public transport, so it was not a major inconvenience; and international travel controls also did not impact a large fraction of the population (both 2).

The COVID-19 mitigation measures have proven challenging around the globe because they require a fundamental shift in human behaviorVan Rooij et al. (2020); Moya et al. (2020). Understanding human behavior and risk perception is central to effective pandemic management, and social and behavioral sciences provide important insights to inform data-driven public policies and strategies to facilitate appropriate interventionsBavel et al. (2020). To estimate risk perceptions of COVID-19 mitigation measures, we draw on the insights from emerging social science research on behavioral mechanisms that shape attitudes, perceptions, and decision-making during the pandemics. Because there is very little published social science research on the current pandemic, our decisions draw from different circumstances and the quality of the data varies (e.g., single studies versus systemic literature reviews, national versus cross-national surveys). Against this background, we predict risk perceptions for each measure based on the absolute levels of severity perception concerning the measures. While individuals may prioritize gains against losses in optimal decision-making (e.g., whether to go out to work and risk infection to earn money or to stay at home and avoid infection), in policy-making, the trade-offs are usually determined at the population levels as a public good Arnot et al. (2020). Therefore, our estimates are overall ratings across populations from different cultures and socioeconomic backgrounds across WEIRD countries. We recognize the need for a more nuanced analysis and variation across countries Moya et al. (2020); Perrotta et al. (2020); however, in the absence of consistent theoretical or empirical justification, we do not consider the effects of individual- or societal-level factors, such as socioeconomic and cultural contexts, laws and regulations, social norms, political beliefs, level of social trust, demographics, intrinsic motivations, and moral support.

The evolution of preferences, knowledge, and the cultural dynamics drive behavioral change Arnot et al. (2020); understanding these helps to inform individual and public decision-making. Considering that people calibrate expectations and behaviors to their specific circumstances, we assume that risk perceptions follow a typical learning curve Fischer et al. (2020): in the first wave, we saw significant behavioral change and adherence at the outbreak when people, responding to fear and the unknown, were motivated to cooperate because both the risk of not adhering and the advantages of adhering became critical for one's own well-being (i.e., perceptions of severe risk). Over time, perceptions were consolidated and moderated with greater population knowledge about the risk and experience with the pandemic (second wave). The third wave saw the introduction of vaccination, but also greater anxiety and trust issues with public health measures and related decrease in public support and overall ratings of efficacy of health instructions. With this in mind, we focus on the second wave, when everyone had initial experience with COVID and was able to consolidate severity perceptions. Policy implications should thus be interpreted with caution.

GDP costs

A complete overview of the cost data used can be found in Table S3.

Details about the multi-objective optimization results

Results of the statistical significance tests are listed in Tables S4, S5, S6, and S7.

Sample intervention plans

All generated intervention plans for 50 different regions can be found on the result's repository JSI (2021). This includes intervention plans for different granularities and for different definitions of NPI's cost, as well as actual NPIs recommended for each specific day.

Fig. S3 and Fig. S4, however, list a few sample results for the reader's convenience – presenting the predicted cost of different plans and their predicted epidemiological curves. From these examples, one can observe that our recommendations consistently outperform the actual implemented intervention plans. In addition, predictions often, but not always, closely match the reality in terms of infection numbers. An extreme example of an error can be found in Fig. 4h. This can happen in cases where the actual NPIs are not very strict and thus the β parameter is high, as in these cases, a small change in the parameter values can lead to a significant difference in the outcome (in most cases, the predicted number of infections is overly pessimistic).

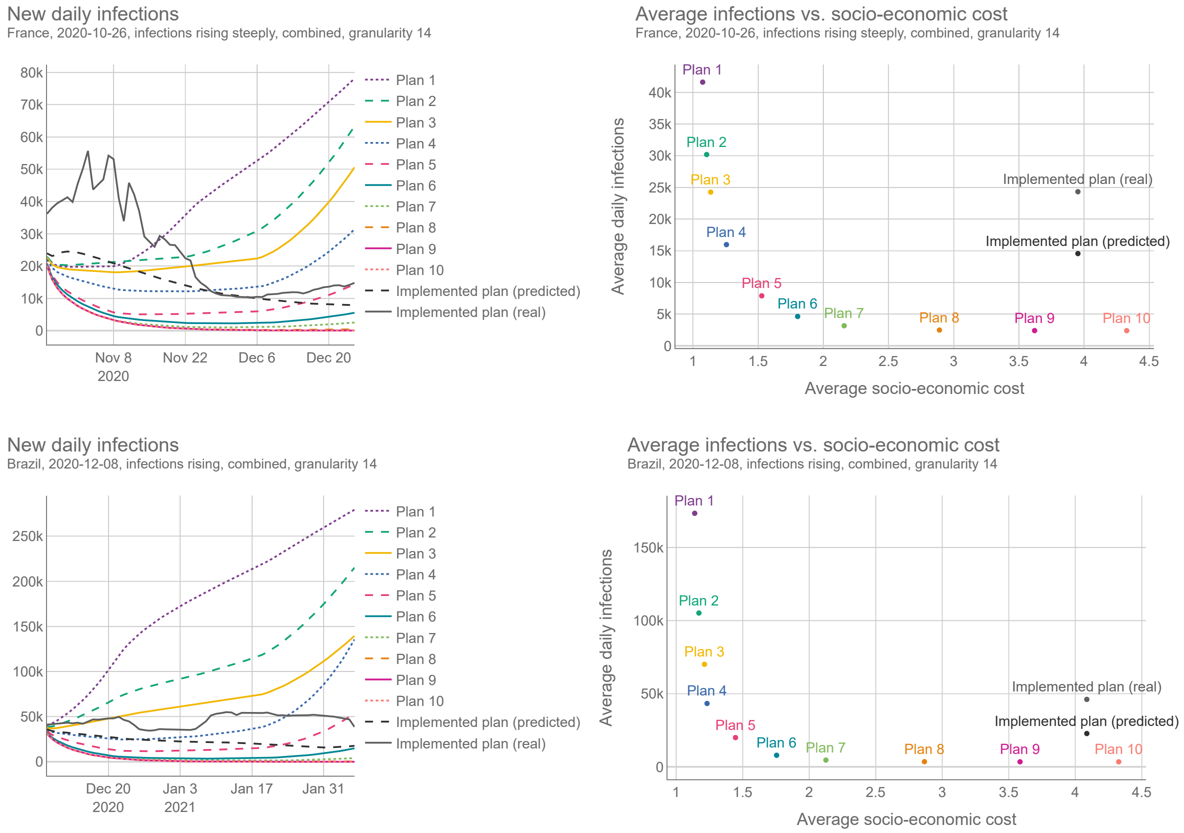

Figure S3. Few examples of the proposed intervention plans. For these plans, we show their predicted number of infections over time (a, c), as well as their trade-off between effectiveness and cost (b, d).

0.1 Comparison of the proposed and implemented solutions

In all 50 test cases, we compared the proposed solutions against the solutions actually implemented in real life (Table S8). Two types of comparisons were made. First, we compared the real solution against a proposed solution that had the same SEC as the implemented one – and then calculated how much the infections were reduced (e.g., if implemented solution had 1000 daily new infections on average, while the

proposed solution with the same cost had 500, then the improvement was 50%). The second comparison was symmetrical, finding a solution with the same number of infections and calculating the improvement in SECs.

When performing this comparison, we had to decide if we should use the real or predicted number of infections for the implemented solution. Both can be argued for, and both yield similar results – so in this comparison, we took the version with fewer infections for each test case, so that it would compare more favourably against our proposed solutions.

The biggest limitation of this comparison is our definition of SECs. The real-life policy-makers were not optimizing for the exact same costs as we had defined, and for different definitions of SECs, the reported results could vary considerably.

Table S1. Description and coding of the health system policies (H).

ID	Description	Coding
H1	Public information campaigns	0 No Covid-19 public information campaign<br>1 Public officials urging caution about Covid-19<br>2 Coordinated public information campaign (e.g., across traditional and social media)
H2	Testing policy	0 No testing policy<br>1 Only those who both (a) have symptoms AND (b) meet specific criteria (e.g., key workers, admitted to hospital, came into contact with a known case, returned from overseas)<br>2 Testing of anyone showing Covid-19 symptoms<br>3 Open public testing (e.g., "drive through" testing available to asymptomatic people)
H3	Contact tracing	0 No contact tracing<br>1 Limited contact tracing; not done for all cases<br>2 Comprehensive contact tracing; done for all identified cases
H6	Facial Coverings	0 No policy<br>1 Recommended<br>2 Required in some specified shared/public spaces outside the home with other people present, or some situations when social distancing not possible<br>3 Required in all shared/public spaces outside the home with other people present or all situations when social distancing not possible<br>4 Required outside the home at all times regardless of location or presence of other people

Table S2. Description and coding of the containment and closure policies (C).

ID	Description	Coding
C1	School and universities closing	0 No policy<br>1 Recommend closing or all schools open with alterations resulting in significant differences compared to non-Covid-19 operations<br>2 Require closing (only some levels or categories, e.g., just high school, or just public schools)<br>3 Require closing all levels
C2	Workplace closing	0 No policy<br>1 Recommend closing (or recommend work from home)<br>2 Require closing (or work from home) for some sectors or categories of workers<br>3 Require closing (or work from home) for all-but-essential workplaces (e.g., grocery stores, doctors)
C3	Cancel public events	0 No policy<br>1 Recommend cancelling<br>2 Require cancelling
C4	Restrictions on gatherings	0 No restrictions<br>1 Restrictions on very large gatherings (the limit is above 1000 people)<br>2 Restrictions on gatherings between 101-1000 people<br>3 Restrictions on gatherings between 11-100 people<br>4 Restrictions on gatherings of 10 people or less
C5	Close public transport	0 No policy<br>1 Recommend closing (or significantly reduce volume/route/means of transport available)<br>2 Require closing (or prohibit most citizens from using it)
C6	Stay at home requirements	0 No policy<br>1 Recommend not leaving house<br>2 Require not leaving house with exceptions for daily exercise, grocery shopping, and 'essential' trips<br>3 Require not leaving house with minimal exceptions (e.g., allowed to leave once a week, or only one person can leave at a time, etc.)
C7	Restrictions on internal movement between cities/regions	0 No policy<br>1 Recommend not to travel between regions/cities<br>2 Internal movement restrictions in place
C8	International travel controls (policy for foreign travellers, not citizens)	0 No restrictions<br>1 Screening arrivals<br>2 Quarantine arrivals from some or all regions<br>3 Ban arrivals from some regions<br>4 Ban on all regions or total border closure

Table S3. Sources for GDP loss for each OxNPI. Assumptions made, the country source analyses, and the NPI stringency in the source country are also listed.

OxNPI	Stringency	Assumption	Country	Source
C1: School closing	3	Closing of all schools	UK	Sadique et al. (2008)
C2: Workplace closing	3	Mandatory closures	US	gdp (2020a)
C3: Cancel public events	2	Cancellation of public events	US	gdp (2020c)
C4: Restrictions on gatherings	2	Same cost as C3	US	gdp (2020c)
C5: Close public transport	1	Closing or significantly reduced	NL	gdp (2020g)
C6: Stay at home requirements	3	Confinement/shelter in place	FR	gdp (2020e)
C7: Restrictions on internal movement	3	Total cessation of domestic tourism	EU	gdp (2020f,d)
C8: International travel controls	3	Travel & tourism restrictions	All	gdp (2020i)
H1: Public information campaigns	2	Communications related to COVID-19	UK	gdp (2020b)
H2: Testing policy	2	Policy of 30 million tests per week	US	Cutler and Summers (2020)
H3: Contact tracing	2	Targeted to contacts of identified cases	US	Cutler and Summers (2020)
H6: Facial coverings	2	EU import of face masks	EU	gdp (2020h)

Table S4. Test statistic values and p-values obtained by the Friedman test for the condensed representation.

Social weights		GDP weights		Combined weights	
$\chi^2(3)$	p	$\chi^2(3)$	p	$\chi^2(3)$	p
≈ 150.678	< 0.01	≈ 119.309	< 0.01	≈ 106.139	< 0.01

Table S5. Test statistic values and p-values obtained by the Friedman test for the full representation.

Social weights		GDP weights		Combined weights	
$\chi^2(3)$	p	$\chi^2(3)$	p	$\chi^2(3)$	p
≈ 192.876	< 0.01	≈ 195.149	< 0.01	≈ 191.473	< 0.01

Table S6. Adjusted p-values resulting from post hoc analysis and the comparison among various values of granularity for the condensed representation. For example, 3 / 0 / 47 in the second row and third column indicates that the optimization with granularity 1 compared to the optimization with granularity 3 performed better for 3 problem instances, equally for 0 problem instances, and worse for 47 problem instances.

Pair	Social weights		GDP weights		Combined weights	
1 vs. 3	< 0.01	3 / 0 / 47	< 0.01	1 / 0 / 49	< 0.01	1 / 0 / 49
1 vs. 7	< 0.01	1 / 0 / 49	< 0.01	4 / 0 / 46	< 0.01	5 / 0 / 45
1 vs. 14	< 0.01	2 / 0 / 48	< 0.01	5 / 0 / 45	< 0.01	5 / 0 / 45
1 vs. 30	< 0.01	5 / 0 / 45	< 0.01	9 / 0 / 41	< 0.01	9 / 0 / 41
3 vs. 7	< 0.01	7 / 0 / 43	< 0.01	5 / 0 / 45	< 0.01	10 / 0 / 40
3 vs. 14	< 0.01	4 / 1 / 45	< 0.01	6 / 0 / 44	< 0.01	8 / 0 / 42
3 vs. 30	< 0.01	8 / 0 / 42	< 0.01	9 / 0 / 41	< 0.01	14 / 0 / 36
7 vs. 14	< 0.01	5 / 1 / 44	≈ 0.07	11 / 0 / 39	< 0.01	13 / 0 / 37
7 vs. 30	< 0.01	12 / 0 / 38	≈ 0.10	12 / 0 / 38	≈ 0.78	22 / 0 / 28
14 vs. 30	≈ 0.06	30 / 1 / 19	≈ 0.10	29 / 2 / 19	< 0.01	31 / 1 / 18

Table S7. Adjusted p-values resulting from post hoc analysis and the comparison among various values of granularity for the full representation. For example, 0 / 0 / 50 in the second row and third column indicates that the optimization with granularity 1 compared to the optimization with granularity 3 performed better for 0 problem instances, equally for 0 problem instances, and worse for 50 problem instances.

Pair	Social weights		GDP weights		Combined weights	
1 vs. 3	< 0.01	0 / 0 / 50	< 0.01	0 / 0 / 50	< 0.01	0 / 0 / 50
1 vs. 7	< 0.01	0 / 0 / 50	< 0.01	0 / 0 / 50	< 0.01	0 / 0 / 50
1 vs. 14	< 0.01	0 / 0 / 50	< 0.01	0 / 0 / 50	< 0.01	0 / 0 / 50
1 vs. 30	< 0.01	0 / 0 / 50	< 0.01	0 / 0 / 50	< 0.01	0 / 0 / 50
3 vs. 7	< 0.01	0 / 0 / 50	< 0.01	0 / 0 / 50	< 0.01	0 / 0 / 50
3 vs. 14	< 0.01	0 / 0 / 50	< 0.01	0 / 0 / 50	< 0.01	0 / 0 / 50
3 vs. 30	< 0.01	0 / 0 / 50	< 0.01	0 / 0 / 50	< 0.01	0 / 0 / 50
7 vs. 14	< 0.01	3 / 0 / 47	< 0.01	2 / 0 / 48	< 0.01	3 / 0 / 47
7 vs. 30	< 0.01	7 / 0 / 43	< 0.01	11 / 0 / 39	< 0.01	2 / 0 / 48
14 vs. 30	≈ 0.31	21 / 0 / 29	≈ 0.38	27 / 0 / 23	< 0.01	9 / 0 / 41

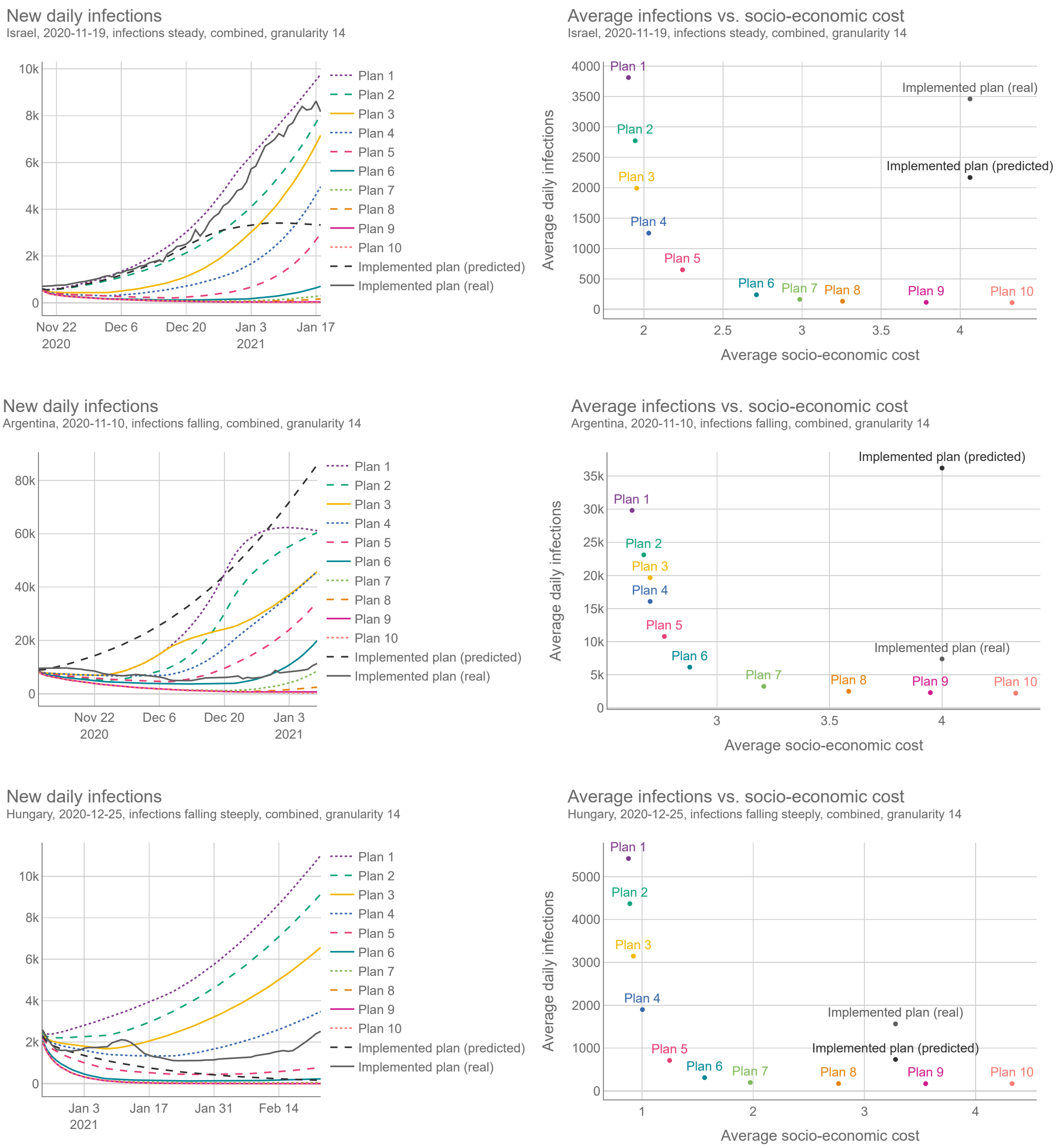

Figure S4. Few examples of the proposed intervention plans. For these plans, we show their predicted number of infections over time (a, c, e), as well as their trade-off between effectiveness and cost (b, d, f).

Territory	Start date	Improvement (SEC) [%]	Improvement (Infections) [%]
Argentina	2020-09-25	72.5	29.2
Argentina	2020-11-10	69.6	29.3
Argentina	2021-01-10	84.1	42.9
Argentina	2021-04-14	69.9	48.0
Belgium	2020-04-01	68.5	64.1
Belgium	2020-06-13	-75.2	-5.3
Brazil	2020-07-18	42.6	22.0
Brazil	2020-12-08	84.9	65.3
Czech Republic	2020-11-18	88.2	68.1
Czech Republic	2021-01-27	63.3	47.5
Czech Republic	2021-03-01	29.2	5.7
France	2020-05-07	-66.8	-29.4
France	2020-10-26	83.5	67.6
France	2020-11-24	83.7	63.5
France	2021-01-19	92.5	55.9
Germany	2020-12-05	82.1	64.6
Germany	2021-03-28	84.9	47.3
Greece	2020-05-05	53.9	8.4
Hungary	2020-10-08	93.1	63.0
Hungary	2020-11-10	73.4	57.6
Hungary	2020-12-25	76.6	62.1
Iran	2020-12-21	85.7	62.0
Israel	2020-09-18	55.1	72.2
Israel	2020-10-19	81.5	65.1
Israel	2020-11-19	94.9	51.8
Israel	2021-03-25	63.2	45.3
Italy	2020-11-01	74.5	60.0
Italy	2020-12-09	86.1	54.0
Italy	2021-01-29	76.8	55.1
Malaysia	2020-05-01	96.6	45.8
Portugal	2020-04-04	-90.0	-30.6
Portugal	2020-10-02	94.8	64.9
Portugal	2021-03-13	78.8	28.4
Slovenia	2020-10-25	88.9	59.8
Slovenia	2021-04-02	86.6	48.9
South Africa	2021-02-14	85.7	51.1
Spain	2020-05-15	94.1	64.3
Spain	2020-08-06	92.7	48.9
Spain	2021-01-18	81.7	48.8
Sweden	2020-05-31	38.9	10.8
Sweden	2020-07-12	80.3	48.2
Sweden	2020-11-13	92.2	62.0
Sweden	2021-03-05	93.9	57.4
Tunisia	2020-12-08	84.8	78.0
Tunisia	2021-05-13	94.5	75.8
United Arab Emirates	2020-09-28	93.3	57.4
United Arab Emirates	2021-03-21	89.6	62.1
United Kingdom	2021-03-27	76.4	50.3
United States	2021-04-14	36.3	51.3
Uruguay	2020-05-29	75.1	34.6
Average		47.1	68.8

Table S8. Relative improvement in the obtained solutions compared to the solution implemented in real life.

REFERENCES

[Dataset] (2020a). Business closures and partial reopenings due to COVID-19 could cost the U.S. trillions. https://news.usc.edu/178979/business-closures-covid-19-pandemic-united-states-gdp-losses/. Last accessed: 2021-10-20

[Dataset] (2020b). Costs of coronavirus advertising. https://www.whatdotheyknow.com/request/costs_of_coronavirus_advertising. Last accessed: 2021-10-20

[Dataset] (2020c). COVID-19 triggered sports and festival cancellations will have staggering economic impact across the country. http://performanceresearch.com/covid-19-triggered-sports-and-festival-cancellations-will-have-staggering-economic-impact-across-the-country. Last accessed: 2021-10-20

[Dataset] (2020d). Domestic tourism expenditure in Europe from 2012 to 2020. https://www.statista.com/statistics/617517/domestic-tourism-expenditure-europe/. Last accessed: 2021-10-20

[Dataset] (2020e). Economic flash France - INSEE estimates the impact of a month of confinement at -3 pts of annual GDP. https://www.tresor.economie.gouv.fr/Articles/2020/03/30/flash-conjoncture-france-l-insee-estime-l-impact-d-un-mois-de-confinement-a-3-pts-de-pib-annuel. Last accessed: 2021-08-16

[Dataset] (2020f). European Union: Gross domestic product (GDP) from 2016 to 2026. https://www.statista.com/statistics/527869/european-union-gross-domestic-product-forecast/. Last accessed: 2021-10-20

[Dataset] (2020g). UITP projects €40bn hit for European public transport in 2020. https://www.railjournal.com/financial/uitp-projects-e40bn-hit-for-european-public-transport-in-2020/. Last accessed: 2021-08-16

[Dataset] (2020h). Which country imported the most face masks? https://ec.europa.eu/eurostat/web/products-eurostat-news/-/ddn-20201006-1. Last accessed: 2021-10-20

[Dataset] (2020i). WTTC research reveals global travel & tourism sector suffered a loss of almost US$4.5 trillion in 2020 due to the impact of COVID-19. https://wttc.org/News-Article/Global-TandT-sector-suffered-a-loss-of-almost-US4-trillion-in-2020. Last accessed: 2021-10-20

Arnot, M., Brandl, E., Campbell, O. L. K., Chen, Y., Du, J., Dyble, M., et al. (2020). How evolutionary behavioural sciences can help us understand behaviour in a pandemic. *Evolution, Medicine, and Public Health* 2020, 264–278

Bavel, J., Baicker, K., and Boggio, P. e. a. (2020). Using social and behavioural science to support COVID-19 pandemic response. *Nature Human Behaviour* 4, 460 – 471

Cutler, D. M. and Summers, L. H. (2020). The COVID-19 Pandemic and the $ 16 Trillion Virus. *JAMA* 324, 1495–1496

Fischer, I., Avrashi, S., Oz, T., Fadul, R., Gutman, K., Rubenstein, D., et al. (2020). The behavioural challenge of the COVID-19 pandemic: indirect measurements and personalized attitude changing treatments (IMPACT). *Royal Society Open Science* 7, 201131

[Dataset] Hale, T., Webster, S., Petherick, A., Phillips, T., and Kira, B. (2020). Oxford COVID-19 government response tracker (OXCGRT)

[Dataset] JSI (2021). Results repository. https://github.com/jsi-dis/ai-covid-interventions

Moya, C., Cruz y Celis Peniche, P., Kline, M. A., and Smaldino, P. E. (2020). Dynamics of behavior change in the COVID world. *American Journal of Human Biology* 32, e23485

Perrotta, D., Grow, A., Rampazzo, F., Cimentada, J., Del Fava, E., Gil-Clavel, S., et al. (2020). Behaviours and attitudes in response to the COVID-19 pandemic: insights from a cross-national Facebook survey. *EPJ Data Science* 17

Sadique, M. Z., Adams, E. J., and Edmunds, W. J. (2008). Estimating the costs of school closure for mitigating an influenza pandemic. *BMC Public Health* 8, 1–7

Van Rooij, B., de Bruijn, A. L., Reinders Folmer, C., Kooistra, E. B., Kuiper, M. E., Brownlee, M., et al. (2020). Compliance with COVID-19 mitigation measures in the United States. *Amsterdam Law School Research Paper*
